# Identification of an 8-miRNA signature as a potential prognostic biomarker for glioma

**DOI:** 10.7717/peerj.9943

**Published:** 2020-09-28

**Authors:** Baowei Ji, Lihua Chen, Qiang Cai, Qiao Guo, Zhibiao Chen, Du He

**Affiliations:** 1 Department of Neurosurgery, Wuhan University, Renmin Hospital, Wuhan, China; 2 Department of Anesthesiology, Wuhan University, Renmin Hospital, Wuhan, China; 3 Department of Oncology, The Central Hospital of Enshi Autonomous Prefecture, Enshi, China

**Keywords:** Glioma, miRNA, Signature, Biomarker

## Abstract

**Background:**

Glioma is the most common form of primary malignant intracranial tumor.

**Methods:**

In the current study, miRNA matrix were obtained from the Chinese Glioma Genome Atlas (CGGA), and then univariate Cox regression analysis and Lasso regression analysis were utilized to select candidate miRNAs and multivariate Cox regression analysis was applied to establish a miRNA signature for predicting overall survival (OS) of glioma. The signature was assessed with the area under the curve (AUC) of the receiver operating characteristic curve (ROC) and validated by data from Gene Expression Omnibus (GEO).

**Results:**

Eight miRNAs (miR-1246, miR-148a, miR-150, miR-196a, miR-338-3p, miR-342-5p, miR-548h and miR-645) were included in the miRNA signature. The AUC of ROC analysis for 1- and 3-year OS in the CGGA dataset was 0.747 and 0.905, respectively. In the GEO dataset, The AUC for 1- and 3-year was 0.736 and 0.809, respectively. The AUC in both the CGGA and GEO datasets was similar to that based on WHO 2007 classification (0.736 and 0.799) and WHO 2016 classification (0.663 and 0.807). Additionally, Kaplan–Meier plot revealed that high-risk score patients had a poorer clinical outcome. Multivariate Cox regression analysis suggested that the miRNA signature was an independent prognosis-related factor [HR: 6.579, 95% CI [1.227−35.268], *p* = 0.028].

**Conclusion:**

On the whole, in the present study, based on eight miRNAs, a novel prognostic signature was developed for predicting the 1- and 3- year survival rate in glioma. The results may be conducive to predict the precise prognosis of glioma and to elucidate the underlying molecular mechanisms. However, further experimental researches of miRNAs are needed to validate the findings of this study.

## Introduction

Glioma is the most prevalent malignant and most aggressive brain tumor (Alexander & Cloughesy, 2017; Molinaro et al., 2019). Standard therapy, which includes surgery followed by radiation and/or chemotherapy, is the most effective treatment strategy for glioma (Lucas Jr et al., 2017). The overall survival rate of glioma is very low, especially the most common subtype: glioblastoma, which 5-year relative survival probability is only limited to 5.1% and median lifespan is only 14.6 months (Bray et al., 2018; Ferlay et al., 2019). Although a phase III trial showed that tumor treating fields plus temozolomide could prolong median overall survival of glioblastoma to 27.2 months (Kim et al., 2020), it is not enough. Theerefore, there is an urgent need to explore more accurate tumor-specific biomarkers for glioma in order further develop novel diagnostic signatures and guide clinical treatment.

MicroRNAs (miRNAs), a class of small non-protein coding RNAs, function through bind them to the 3′-UTRs (3′-untranslated regions) of target mRNAs to degrade mRNAs or negatively regulate the expression of target proteins (Xiong et al., 2018; Zhang et al., 2019). Previous cell experiments have demonstrated that miRNAs are expressed in many cancers abnormally, including glioma, and play an pivotal role in the regulating of different biological processes, such as cell proliferation, invasion, metastasis and apoptosis (Boos et al., 2019). For example, a study reported that, in glioma, miR-139e3p was expressed abnormally, and could suppress cell proliferation invasion and migration (Tian et al., 2019).

Advances in omics technology have provide new strategies for the understanding, and for the diagnosis and treatment of cancer systematically (Casuscelli et al., 2017). Recently, RNA-Seq profiling has been developed for the identification of novel molecular markers and mechanisms in numerous tumors, such as pancreatic adenocarcinoma and clear cell renal cell carcinoma (Feng et al., 2018; Luo et al., 2019).

Similar to most malignant tumors, glioma derives from genetic and epigenetic alterations (Wang et al. 2019). However, a number of researches have primarily focused on coding genes. Few researches have been studied to date on the functions and prognostic value of miRNAs in glioma, at least to the best of our knowledge. Herein, the original matrix files of glioma, collected from the Chinese Glioma Genome Atlas (CGGA; http://www.cgga.org.cn) were analyzed to identify a prognostic miRNA signature. The data were then validated by data from Gene Expression Omnibus (GEO; https://www.ncbi.nlm.nih.gov/geo/). Moreover, functional enrichment analysis of target genes of miRNAs was performed to explore the underlying molecular mechanisms of miRNAs in glioma. The findings of this study may be conducive to the identification the prognosis- related miRNAs in glioma and shed light onto the molecular mechanisms of glioma.

## Materials and Methods

### Patient datasets

The raw data of miRNA expression and relevant clinical characteristics (age, gender, WHO grade, 1p/19q codel, IDH mutation, overall survival and censor status) were downloaded from the CGGA and GEO databases (GSE25632 and GSE104554 datasets). The platform for the CGGA dataset and GSE25632 was GPL8179 (Illumina Human v2 MicroRNA expression beadchip), and for GSE104554 it was GPL14613 (Affymetrix Multispecies miRNA-2 Array). Perl 5.0 (http://www.perl.org/) was used to background correction and normalization of all miRNA expression. Patients lack of pathologic diagnosis and corresponding survival time and survival state will be removed. The overall survival time was measured from the day that patients were diagnosed as glioma to death or last observation (Nov.28, 2019). The censor mark: 1 in CGGA database represented that patients have died at last clinical assessment, and censor mark: 0 represented that patients were still alive at last clinical assessment. The grade classification of glioma was based on WHO 2007 criteria which classifies glioma patients into four subtypes, and WHO 2016 criteria which mainly classifies diffuse glioma into five subtypes.

### Identification and validation of miRNA signature

Univariate Cox regression analysis and Lasso regression analysis were performed to analyze miRNA expression from CGGA dataset to select overall survival (OS) associated-miRNAs. Subsequently, multivariate Cox regression analysis was used to determine top survival-related candidate miRNAs and develop a risk score formula which divided patients into two subgroups (low- and high- risk groups); risk score = coef * the expression of miRNA_1_ + coef * the expression of miRNA_2_+ coef * the expression of miRNA_3_+ ……+ coef * the expression of miRNA_x_. The receiver operating characteristic curve (ROC) was pictured to assess the predicted efficiency of this miRNA signature. In addition, data from GEO was used to validate this result.

### Bioinformatics analysis of target genes

Target genes of candidate miRNAs were predicted using two tools: miRtarbase (http://mirtarbase.cuhk.edu.cn/php/index.php) (Chou et al., 2018) and TarBase v.8 (http://carolina.imis.athena-innovation.gr) (Paraskevopoulou, Vlachos & Hatzigeorgiou, 2016). The overlapping genes were then used for further analysis.

### Functional enrichment analysis and PPI network of target genes

Kyoto Encyclopedia of Genes and Genomes (KEGG) and Gene Ontology (GO) enrichment analysis were performed using the Database for Annotation, Visualization and Integrated Discovery DAVID; https://david.ncifcrf.gov) database (Huang da, Sherman & Lempicki, 2009) with FDR <  0.05 being set as criterion. In addition, a PPI network of target genes was established with Search Tool for the Retrieval of Interacting Genes (STRING; https://string-db.org) (Szklarczyk et al., 2017), and then processed by Cytoscape (http://www.cytoscape.org).

### Statistical analysis

All clinicopathological data are presented as number (No.) and percentage (%). For categorical data, the differences among different groups were compared using a Chi-square test, whereas measurement data were compared using a *t*-test or one-way ANOVA. Univariate Cox regression analysis and Lasso regression analysis were applied to screen out OS-related miRNAs, and multivariate Cox regression analysis were utilized for establishing miRNA signature. Moreover, univariate and multivariate Cox regression analysis were also applied for the identification of independent OS-related factors. In addition, the Kaplan–Meier method and log-rank test were applied to form survival curves and compare survival differences. All statistical analyses were performed with R (version 3.2.3, Vienna, Austria). *P* < 0.05 was considered to indicate statistical differences.

## Results

### Patient data sets

In the current study, a total of 311 glioma patients (190 samples in the CGGA dataset and 121 samples in the GEO dataset) were enrolled. The detailed baseline characteristics of all patients were presented in Table S1.

### Identification and validation of miRNA signature

**Figure 1:**
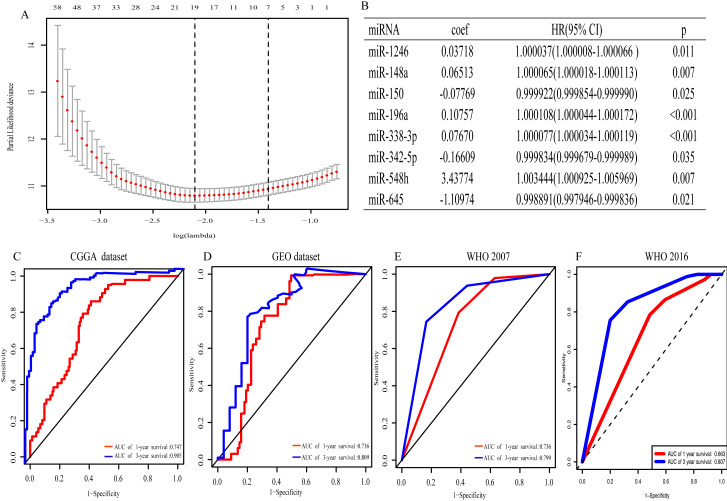
The eight- miRNA signature. (A) “Leave- one-out- cross-validation” for parameter selection in LASSO regression; (B) eight miRNAs identified in multivariate Cox regression; (C) the area under the receiver operating characteristic (ROC) curve (AUC) for 1- and 3-year overall survival of glioma patients in CGGA dataset; (D) in GEO dataset; (E) based on WHO 2007 classification; (F) based on WHO 2016 classification.

With univariate regression analysis, 690 miRNAs were selected in the CGGA dataset, and they were further analyzed by Lasso regression analysis, which screened out 19 miRNAs (Fig. 1A). Finally, multivariate Cox regression analysis was utilized to construct an eight-miRNA signature (miR-1246, miR-148a, miR-150, miR-196a, miR-338-3p, miR-342-5p, miR-548 h and miR-645) (Fig. 1B). The risk score was calculated and classified patients into two subgroups. The AUC (the area under the curve) of this miRNA signature for predicting 1- and 3-year OS in the CGGA dataset was 0.747 and 0.905, respectively (Fig. 1C), similar to the AUC of GEO dataset (0.736 and 0.809) (Fig. 1D). Consistent with the AUC in both the CGGA and GEO datasets, the AUC for 1- and 3-year OS of WHO 2007 classification was 0.736 and 0.799, respectively (Fig. 1E), and of WHO 2016 classification was 0.663 and 0.807, respectively (Fig. 1F). The distribution of the characteristics of the eight-miRNA signature in CGGA dataset and GEO dataset are illustrated in Fig. S1.

### Association between miRNA signature and clinicopathological characteristics

**Figure 2:**
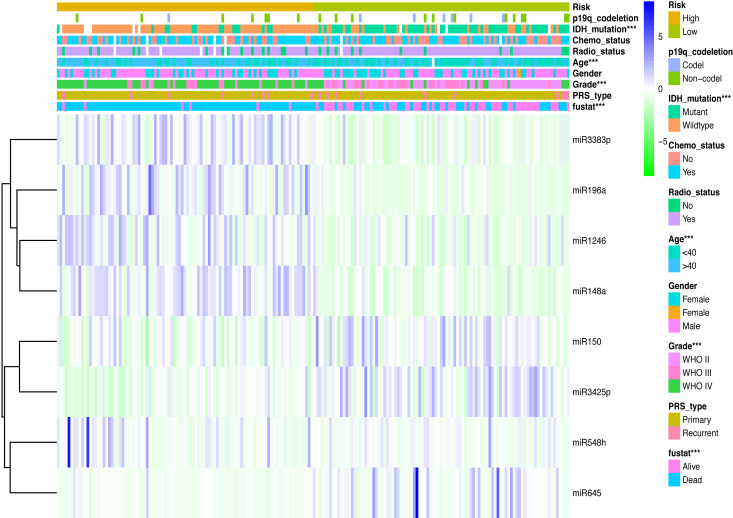
Comparison of clinical parameter between low- risk score and high- risk score patients. Comparison of clinical parameter between low- risk score and high- risk score patients. ^∗∗∗^*p* < 0.001.

**Figure 3:**
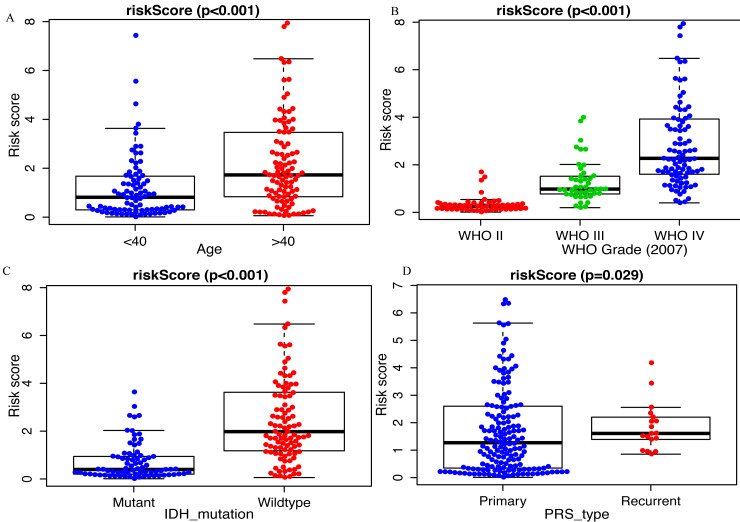
Comparison of risk score among different clinicopathological characteristics. Comparison of risk score between (A) age <40 and age >40 patients, (B) different WHO grade, (C) IDH mutant and IDH wildtype patients, (D) primary tumor and recurrent tumor.

Clinicopathological data including age, sex, tumor grade, recurrent, radiotherapy, chemotherapy, IDH mutation and 1p19q_codeletion were collected. There was a significant difference in age (<0.001), grade (<0.001) and IDH mutation status (<0.001) between the high- and low-risk groups (Fig. 2). In addition, in patients with an age >  40 years, the risk score was significantly higher than that in patients with an age <40 years (Fig. 3A, *P* < 0.001). A similar result was observed with tumor recurrence in patients (Fig. 3D, *P* = 0.029). Moreover, when compared with patients with IDH mutation, the risk score was significantly increased in patients with IDH wild-type (Fig. 3C, *P* < 0.001). In addition, the risk score increased with the increasing WHO 2007 grade (Fig. 3B, *P* < 0.001). In addition, risk scores in different grades of WHO 2016 grade were also significant difference (Fig. S2). Meanwhile, the expression levels of miR-148a, miR-196a, miR-1246 and miR-338-3p were up-regulated in patients with IDH mutation, whereas, miR-645 and miR-342-5p expression levels were down-regulated (Fig. S2). The expression of miR-148a was up-regulated in patients with tumor recurrence, those receiving chemotherapy and in those aged >40 years (Fig. S3). The same phenomenon was observed for miR-1246 in patients receiving chemotherapy and in those aged >40 years (Fig. S3). miR-196a and miR-338-3p were also upregulated in patients aged >40 years (Fig. S3). A significant difference was observed in miR-148a, miR-150, miR-1246, miR-338-3p, miR-196a, miR-645 and miR-342-5p expression among the different WHO 2007 grades (Fig. S3).

### The miRNA signature is an independent OS-related factor

**Figure 4:**
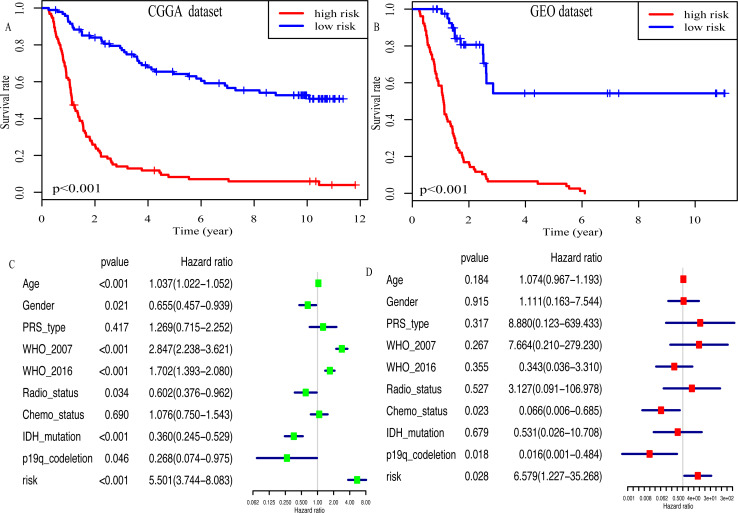
Risk score as an independent prognostic factor. (A) Kaplan–Meier plot of risk score in CGGA dataset. (B) in GEO dataset. (C) The result of univariate Cox regression analysis in the CGGA dataset. (D) The result of multivariate Cox regression analysis in the CGGA dataset.

Kaplan–Meier plot was depicted to show the relationship between the prognosis of glioma and the risk score, along with the expression levels of eight miRNAs. The results indicated that the prognosis of high-risk patients was worse than that of low-risk patients (*P* < 0.001, Fig. 4A). Similar results were observed in the GEO dataset (*P* < 0.001, Fig. 4B). Moreover, univariate Cox regression analysis suggested that risk score was highly associated with the OS of glioma patients (HR, 5.501; 95% CI [3.744–8.083]; *P* < 0.001; Fig. 4C). Multivariate Cox regression analysis identified that this miRNA signature was an independent OS-related factor (HR: 6.579, 95% CI (1.227–35.268), *p* = 0.028, Fig. 4D).

**Figure 5:**
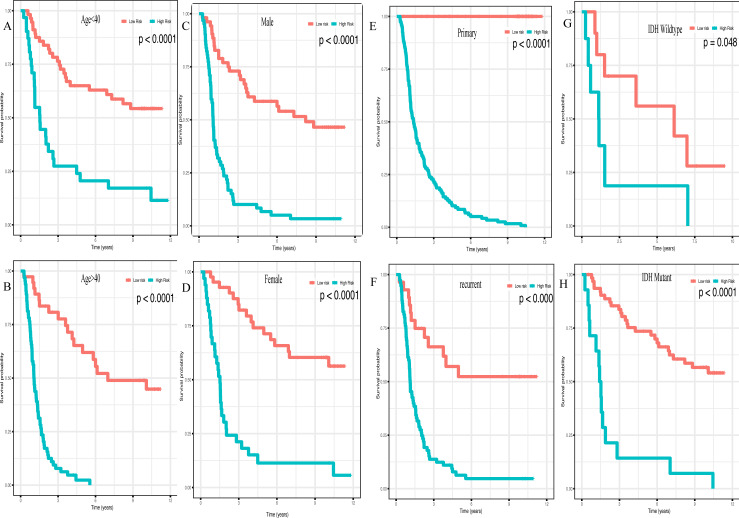
Survival analysis of low- and high- risk score patients among different clinicopathological subgroups. Survival analysis of low- and high- risk score patients in (A) age < 40 group, (B) age > 40 group, (C) male group, (D) female group, (E) primary tumor group, (F) recurrent tumor group, (G) IDH wildtype group, (H) IDH mutant group.

**Figure 6:**
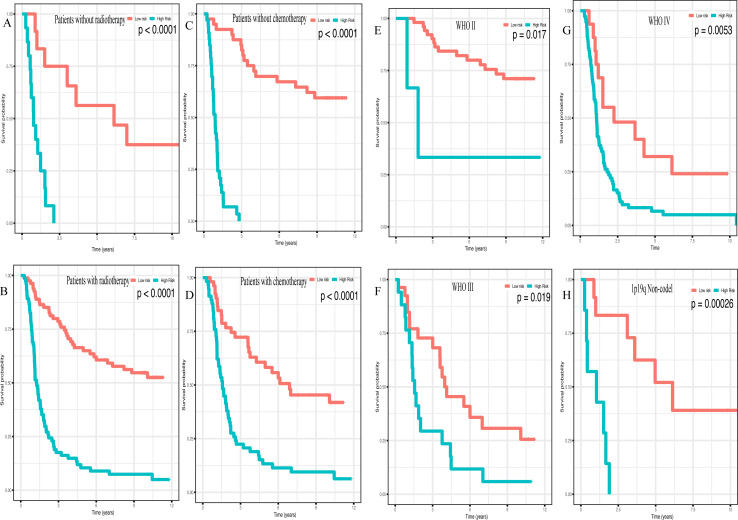
Survival analysis of low- and high- risk score patients among different clinicopathological subgroups. Survival analysis of low- and high- risk score patients in (A) radiotherapy group, (B) without radiotherapy group, (C) chemotherapy group, (D) without chemotherapy group, (E) WHO II group, (F) WHO III group, (G) WHO IV group, (H) 1p19q Non-codel group.

In addition, high risk patients had a poorer clinical outcome in all age groups (<40 group (Fig. 5A, *P* < 0.001), >40 group (Fig. 5B, *P* < 0.001)). A high risk score was also associated with a poorer clinical outcome among males (Fig. 5C, *P* < 0.001), females (Fig. 5D, *P* < 0.001), and as regards primary tumor (Fig. 5E, *P* < 0.001), tumor recurrence (Fig. 5F, *P* < 0.001), IDH wild-type (Fig. 5G, *P* < 0.048), IDH mutation (Fig. 5H, *P* < 0.001), radiotherapy treatment (Fig. 6A, *P* < 0.001), no radiotherapy treatment (Fig. 6B, *P* < 0.001), chemotherapy treatment (Fig. 6C, *P* < 0.001), no chemotherapy treatment (Fig. 6D, *P* < 0.001), WHO II grade (Fig. 6E, *P* = 0.017), WHO III grade (Fig. 6F, *P* = 0.019), WHO IV grade (Fig. 6G, *P* = 0.005) and 1p19q Non-codel (Fig. 6H, *P* < 0.001).

Moreover, the high expression level of miR-148a (*P* < 0.001), miR-196a (*P* < 0.001), miR-1246 (*P* < 0.001), miR-338-3p (*P* < 0.001) along with a low expression level of miR-150 (*P* < 0.001), miR-645 (*P* < 0.001) and miR-342-5p (*P* < 0.001) predicted poorer outcome in glioma (Fig. S4).

### Functional enrichment analysis and PPI network

**Figure 7:**
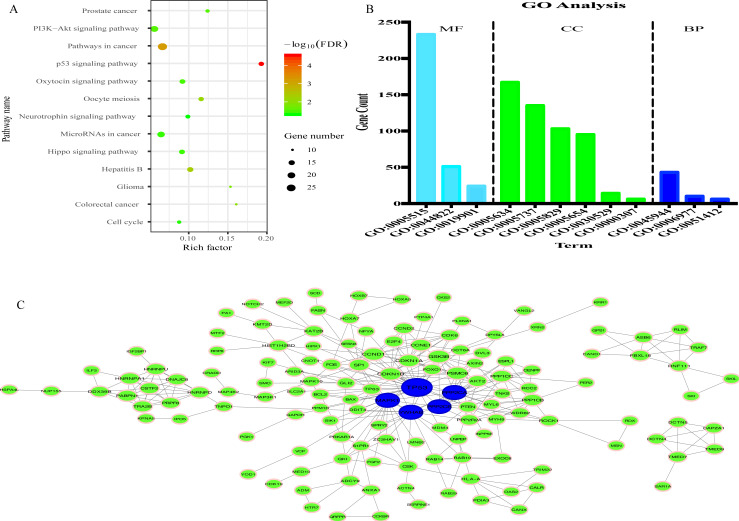
Prediction of functions of eight miRNAs. (A) Kyoto Encyclopedia of Genes and Genomes (KEGG) analysis of target genes. (B) Gene Ontology (GO) analysis of target genes. (C) Protein-protein Interaction (PPI) network. Nodes represent target gene with confidence >0.9. Blue nodes represent hub genes. Edges represent the interaction among target genes.

335 genes targeted by 8 miRNAs were predicted (Fig. S5). KEGG analysis results revealed that 335 target genes were mainly involved in 13 pathways (Fig. 7A). In addition, 12 GO terms were enriched, including 3 biological process, 4 cellular component and 3 molecular function (Fig. 7B).

To explore the interplay among the 335 genes, a PPI network was constructed using the STRING tool with confidence >0.9 as a cut-off criterion, and this was then processed by Cytoscape (Fig. 7C). Moreover, in the PPI network, TP53, MAPK1, YWHAE, PPP2CA and PPP2CB were the significant nodes as they had the most connections with other nodes.

## Discussion

Glioma is the most common brain tumor caused by numerous carcinogenic factors (Alarcón et al., 2019). A previous study demonstrated that miRNAs, approximately 22 nt in length without a protein-coding capacity, affect the occurrence of glioma by interacting with proto-oncogenes and/or tumor suppressor genes (Miroshnichenko & Patutina, 2019). However, a single miRNA is of limited value for the diagnosis and prognosis of glioma. Hence, it is of utmost importance to explore multiple miRNA signatures in glioma, which will help to illustrate the mechanisms of glioma and may provide an effective and precise prediction of the prognosis for patients with glioma.

In the present study, miRNA profiles, which were downloaded from the CGGA database were used to construct a prognostic miRNA signature. Ultimately, eight miRNAs (miR-1246, miR-148a, miR-150, miR-196a, miR-338-3p, miR-342-5p, miR-548 h and miR-645) were identified and utilized to form a risk score formula with multivariate Cox regression analysis. Kaplan–Meier analysis revealed that seven miRNAs (miR-148a, miR-196a, miR-1246, miR-338-3p, miR-150, miR-645 and miR-342-5p) were closely related with the prognosis of glioma patients. miR-148a has been found to be abnormally expressed in diverse malignant tumors, such as colorectal cancer (Tsai et al., 2019), ovarian cancer (Zhu et al., 2019), hepatocellular carcinoma (Babu & Muckenthaler, 2019), breast cancer (Lacerda et al., 2019), esophageal cancer (Wang et al., 2019), gastric cancer (Song et al., 2018), non-small cell lung cancer (Chu et al., 2018) and glioma (Cai, Zhu & Gong, 2018; Cui et al., 2017). In addition, Cai, Zhu & Gong (2018) found that miR-148a regulated STAT3 pathway activity by directly targeting CADM1 to promote glioma cell growth and metastasis. Furthermore, miR-148a has been shown to promote migration and invasion by downregulating the tumor suppressor gene, GADD45A, in glioma cells (Cui et al., 2017). In addition, miR-150 has been shown to be associated with the prognosis of a number of types of cancer and to decrease the proliferation and migration of glioma (Sakr et al., 2016). Consistent with the findings of this study, a previous study demonstrated glioma patients with a higher miR-196a expression had a poorer clinical outcome (Guan et al., 2015). In some malignant tumors, the tumor-suppressor gene miR-338-3p is expressed at low levels. Additionally, Shang et al. (2016) found miR-338-3p was an important prognostic factor in glioma and regulated the malignant biological behaviors of glioma cells by suppressing MACC1 expression. However, there is limited information available on the function of miR-1246, miR-342-5p, miR-548 h and miR-645 in glioma. It has been reported that miR-1246 increases the stemness and invasiveness of non-small cell lung cancer (Kim et al., 2016; Lin et al., 2019). miR-645 has been shown to be significantly associated with the poor prognosis of patients with head and neck cancer, and to promote head and neck cancer cell invasion and migration (Sun et al., 2015). In addition, the overexpression of miR-342-5p affects HER2 breast cancer cell motility and mitochondrial stability (Lindholm et al., 2019).

On the basis of risk score, patients in the present study were divided into the high- and low- risk group. The association between risk score and clinicopathological characteristics was also determined. Risk score was positively associated with age and WHO classification, suggesting that the eight miRNAs may play a vital role in the process of tumorigenesis and in the development of glioma. Moreover, Kaplan–Meier analysis revealed that the high-risk patients had a more unfavorable OS. In addition, multivariate Cox regression analysis identified that this miRNA signature was an independent prognosis- related factor. The AUC of the prognostic eight-miRNA signature in the CGGA dataset (1-year, 0.747; 3-year, 0.905) was close to that in the GEO dataset (1-year, 0.736; 3-year, 0.809) and WHO classification (1-year, 0.736; 3-year, 0.799), indicating that the predicting efficiency of this miRNA signature was precise and it was suitable for the prediction of 1- and 3-year OS in glioma.

To further explore the biological function of the eight miRNAs, the present study predicted 335 mRNAs targeted by the eight miRNAs using two tools: miRtarbase and TarBase v.8. In KEGG analysis, 13 pathways were enriched, including a few pathways which were markedly associated with the occurrence and development of tumors. For example, the PI3K/Akt signaling pathway is activated by a number of types of cellular stimulation and is often genetically altered in human cancers; it is also an important regulator of fundamental cellular processes, such as transcription, translation, proliferation and apoptosis (Alzahrani, 2019). It has been proven that the activation of the PI3K/Akt signaling pathway promotes the proliferation, metabolism, migration and angiogenesis of glioma (Fan & Weiss, 2010). The p53 signaling pathway is another vital pathway that regulates cellular processes, cell growth and death. The activation of the p53 signaling pathway is induced by a series of stress signals, including oxidative stress and DNA damage (Yi et al., 2019). In addition, in the present study, GO analysis showed that 335 target genes were mainly related to 12 terms. All the results of GO and KEGG analysis revealed that the eight candidate miRNAs played an important role in the oncogenesis and progression of glioma.

The eight-miRNA signature showed an excellent prediction ability for glioma patients. However, there are still several limitations that need to be improved. Firstly, as all patient information was gathered from public databases, the possibility of selection bias cannot be eliminated. Secondly, due to some participants who did not experience the event of death on the date of their last follow-up, the Lost-to-follow-up bias may not be eliminated. Thence, we discarded patients without corresponding follow-up time and survival state at last clinical assessment to Lost-to-follow-up bias. Finally, no experimental studies were performed to validate the functions of the eight miRNAs in glioma, particularly miR-1246, miR-342-5p, miR-548 h and miR-645. Thus, further basic experiments are warranted to validate the findings of the present study.

In summary, in the present study, an eight-miRNA signature was identified, which was an independent prognosis- related factor for glioma patients. These results may contribute to a better understanding of glioma at a molecular level. However, further basic experiments of miRNAs are required to validate the findings in the present study.

##  Supplemental Information

10.7717/peerj.9943/supp-1 Supplemental Information 1 The baseline characteristics of glioma patients in CGGA and GEO dataset

10.7717/peerj.9943/supp-2 Supplemental Information 2 Distribution of risk score, survival status and expression profile of eight miRNAs (A) in CGGA dataset, (B) in GEO dataset.

10.7717/peerj.9943/supp-3 Supplemental Information 3 Comparison of risk score between different WHO 2016 grade 1, 2, 3, 4 and 5 represent diffuse or anaplastic astrocytomas with IDH-mutant; oligodendroglioma or anaplastic oligodendroglioma with IDH-mutant and 1p19q co-deleted; diffuse or anaplastic astrocytomas with IDH-wild type; glioblastoma with IDH-mutant and glioblastoma with IDH-wild type, respectively.

10.7717/peerj.9943/supp-4 Supplemental Information 4 Comparison of expression of eight miRNAs between IDH mutant and IDH wildtype patients

10.7717/peerj.9943/supp-5 Supplemental Information 5 Comparison of expression of eight miRNAs

10.7717/peerj.9943/supp-6 Supplemental Information 6 Survival analysis of eight miRNAs

10.7717/peerj.9943/supp-7 Supplemental Information 7 A miRNA- target gene network Green triangle and red node represent miRNA and target gene, respectively.

## Abbreviations

CGGA: Chinese Glioma Genome Atlas
GEO: Gene Expression Omnibus
DEGs: differentially expressed genes
DAVID: Database for Annotation, Visualization and Integrated Discovery
STRING: Search Tool for the Retrieval of Interacting Genes
AUC: the area under the curve
ROC: the receiver operating characteristic curve
PPI: protein-protein interaction
MCODE: Molecular Complex Detection
KEGG: Kyoto Encyclopedia of Genes and Genomes
GO: Gene Ontology

## Additional Information and Declarations

### Data Availability

The following information was supplied regarding data availability: All data were gathered from the CGGA dataset (Chinese Glioma Genome Atlas, microRNA_198; http://www.cgga.org.cn/download.jsp) and GEO: GSE25632.
