# Supplementary material for: Identification of an 8-miRNA signature as a potential prognostic biomarker for glioma
Source: PeerJ. 2020 Sep 28;8:e9943. doi: 10.7717/peerj.9943 (PMC7528815; doi:10.7717/peerj.9943)
Supplement: Supplemental Information 1 [file peerj-08-9943-s001.docx]

Table SI. The baseline characteristics of glioma patients in CGGA and GEO dataset.

| **Parameter** | **CGGA dataset** | **GEO dataset** | **p** |
| --- | --- | --- | --- |
| Age (Years) |  |  | <0.001 |
| <40 | 91(47.9%) | 31(25.6%) |  |
| >40 | 99(52.1%) | 90(74.4%) |  |
| Gender |  |  | 0.485 |
| Male | 115(60.5%) | 78(64.5%) |  |
| Female | 75(39.5%) | 43(35.5%) |  |
| Grade |  |  | 0.263 |
| WHO II | 55(28.9%) | 25(20.7%) |  |
| WHO III | 44(23.2%) | 32(26.4%) |  |
| WHO IV | 91(47.9%) | 64(52.9%) |  |
| PRS_type |  |  | 0.910 |
| Primary | 172(90.5%) | 110(90.9%) |  |
| Recurrent | 18(9.5%) | 11(9.1%) |  |
| Radiotherapy |  |  |  |
| No | 27 | NA |  |
| Yes | 156 | NA |  |
| NA | 7 | 121 |  |
| Chemotherapy |  |  |  |
| No | 73 | NA |  |
| Yes | 111 | NA |  |
| NA |  | 121 |  |
| IDH mutation |  |  |  |
| Wildtype | 103 | NA |  |
| Mutant | 77 | NA |  |
| NA | 10 | 121 |  |
| 1p19q_codeletion |  |  |  |
| Non-codel | 19 | NA |  |
| Codel | 7 | NA |  |
| NA | 164 | 121 |  |
| Survival status |  |  | 0.384 |
| Alive | 59(31.1%) | 32(26.4%) |  |
| Dead | 131(68.9%) | 89(73.6%) |  |
| Risk score |  |  | 0.018 |
| Low | 95(50%) | 44(36.4%) |  |
| High | 95(50%) | 77(63.6%) |  |
| Total | 190(100%) | 121(100%) |  |
