# Supplementary figures and images for: Identification of an 8-miRNA signature as a potential prognostic biomarker for glioma
Source: PeerJ. 2020 Sep 28;8:e9943. doi: 10.7717/peerj.9943 (PMC7528815; doi:10.7717/peerj.9943)

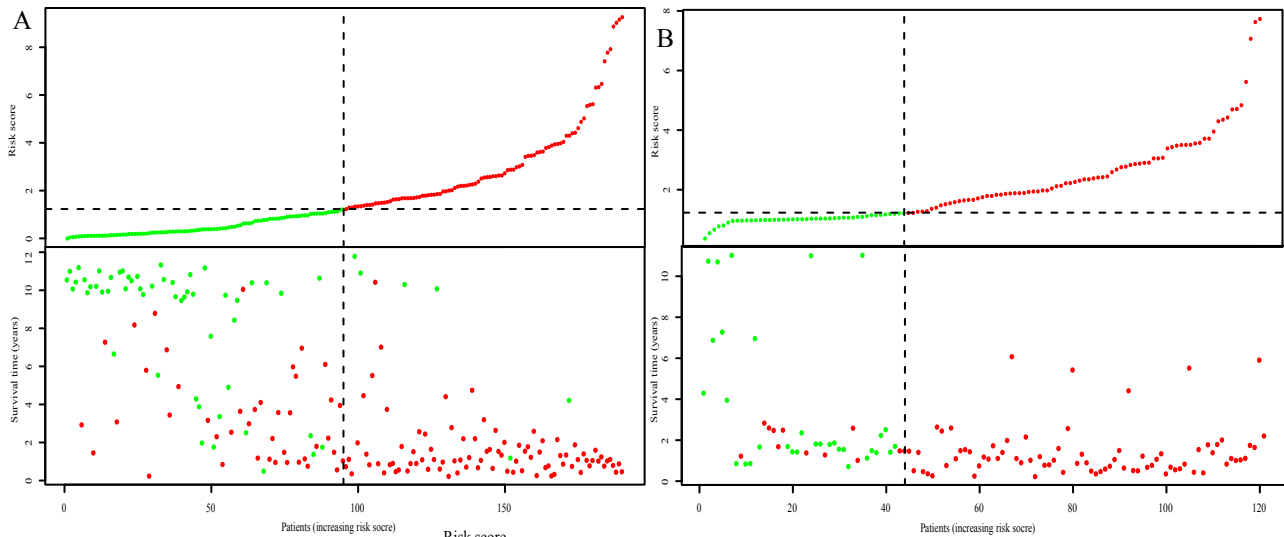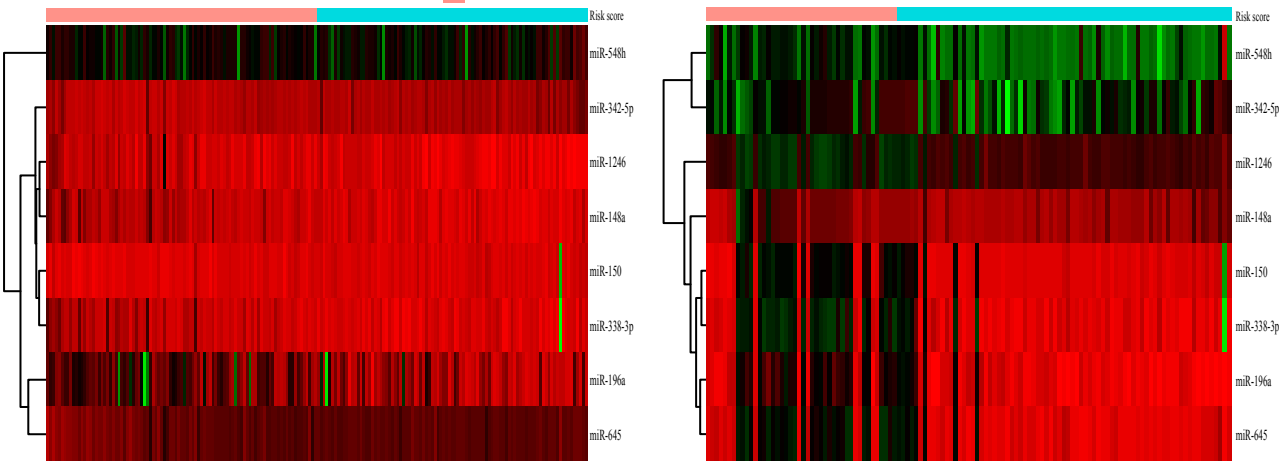

Supplement: Supplemental Information 2 — (A) in CGGA dataset, (B) in GEO dataset. [file peerj-08-9943-s002.pdf]

**riskScore (p=1.208e-10)**

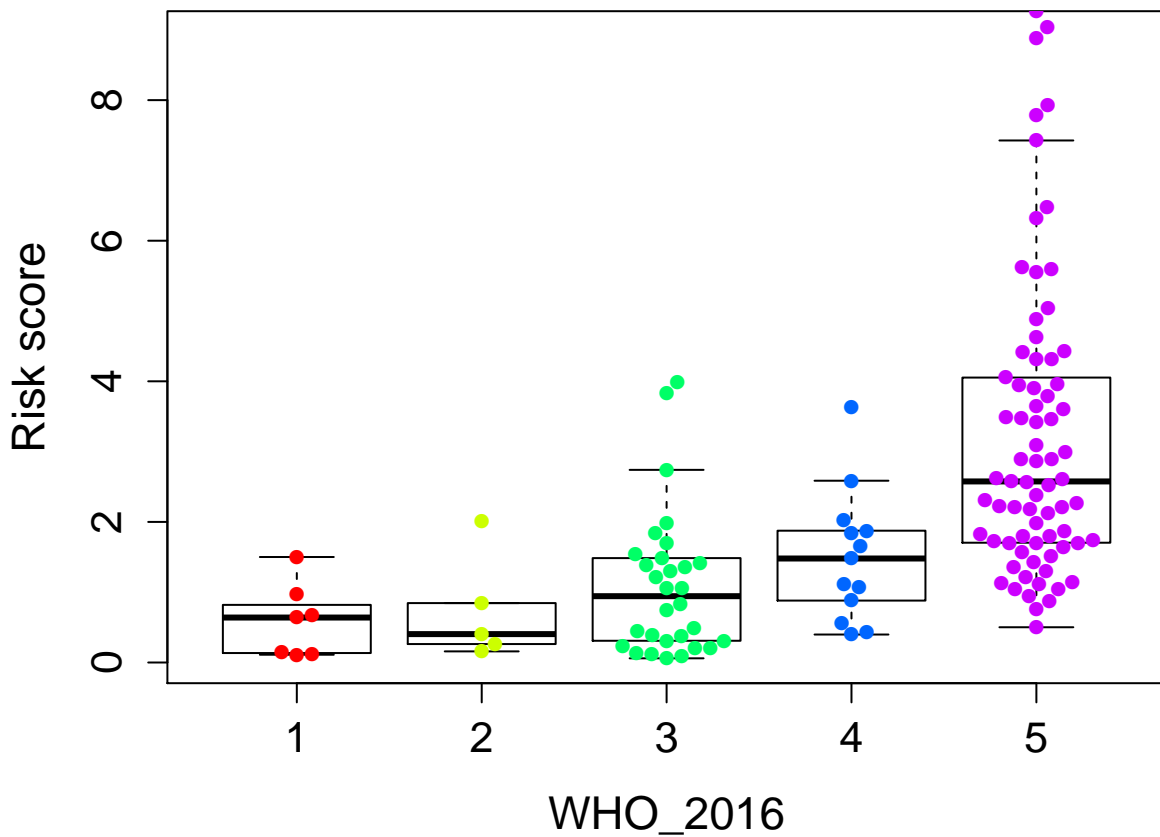

Supplement: Supplemental Information 3 — 1, 2, 3, 4 and 5 represent diffuse or anaplastic astrocytomas with IDH-mutant; oligodendroglioma or anaplastic oligodendroglioma with IDH-mutant and 1p19q co-deleted; diffuse or anaplastic astrocytomas with IDH-wild type; glioblastoma with IDH-mutant and glioblastoma with IDH-wild type, respectively. [file peerj-08-9943-s003.pdf]

miR-148a ( $P=1.436\text{e-}15$ )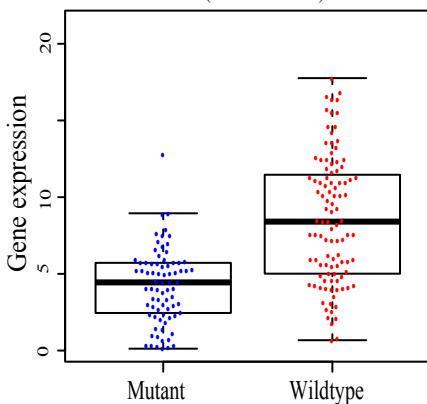miR-196a ( $P=5.302\text{e-}09$ )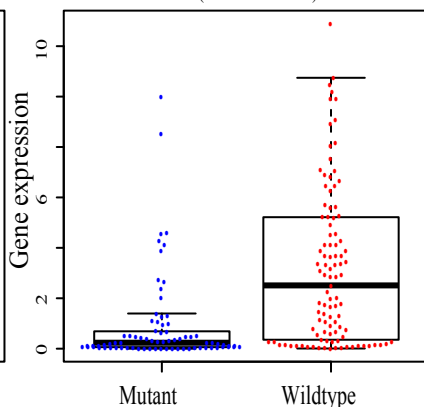miR-645 ( $P=0.025$ )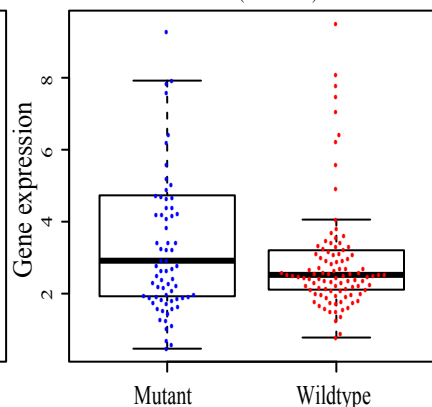miR-1246 ( $P=2.553\text{e-}08$ )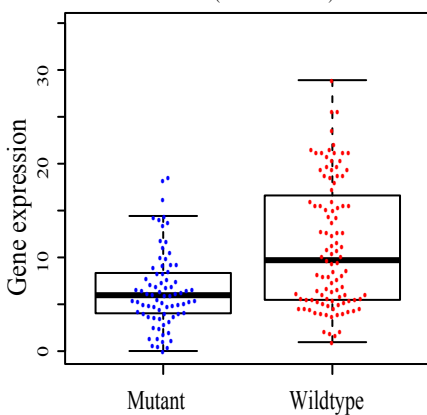miR-338-3p ( $P=2.176\text{e-}04$ )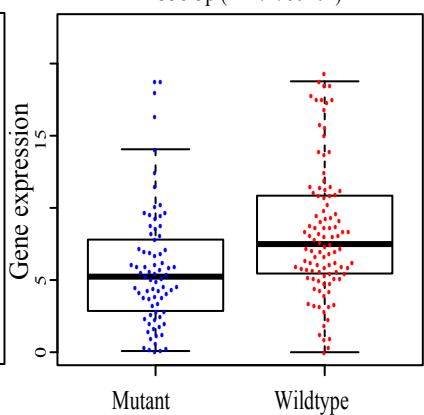miR-342-5p ( $P=3.995\text{e-}06$ )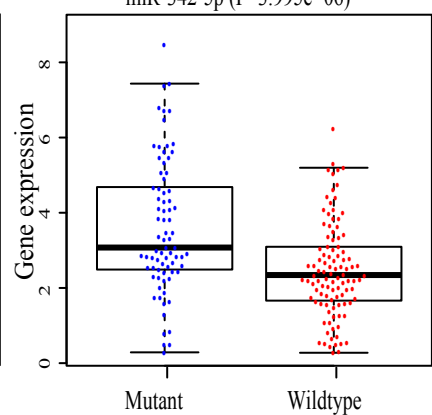

Supplement: Supplemental Information 4 [file peerj-08-9943-s004.pdf]

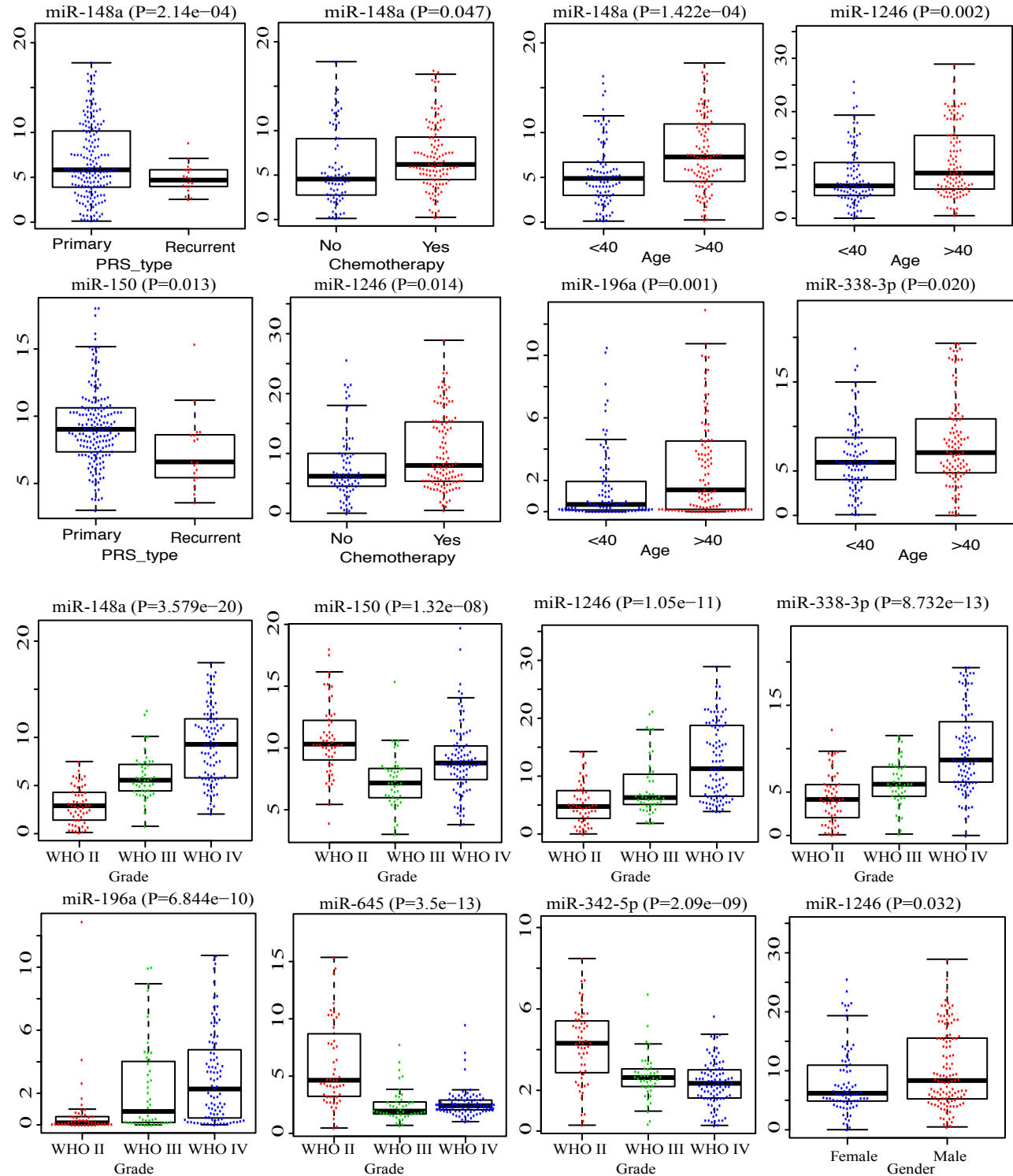

Supplement: Supplemental Information 5 [file peerj-08-9943-s005.pdf]

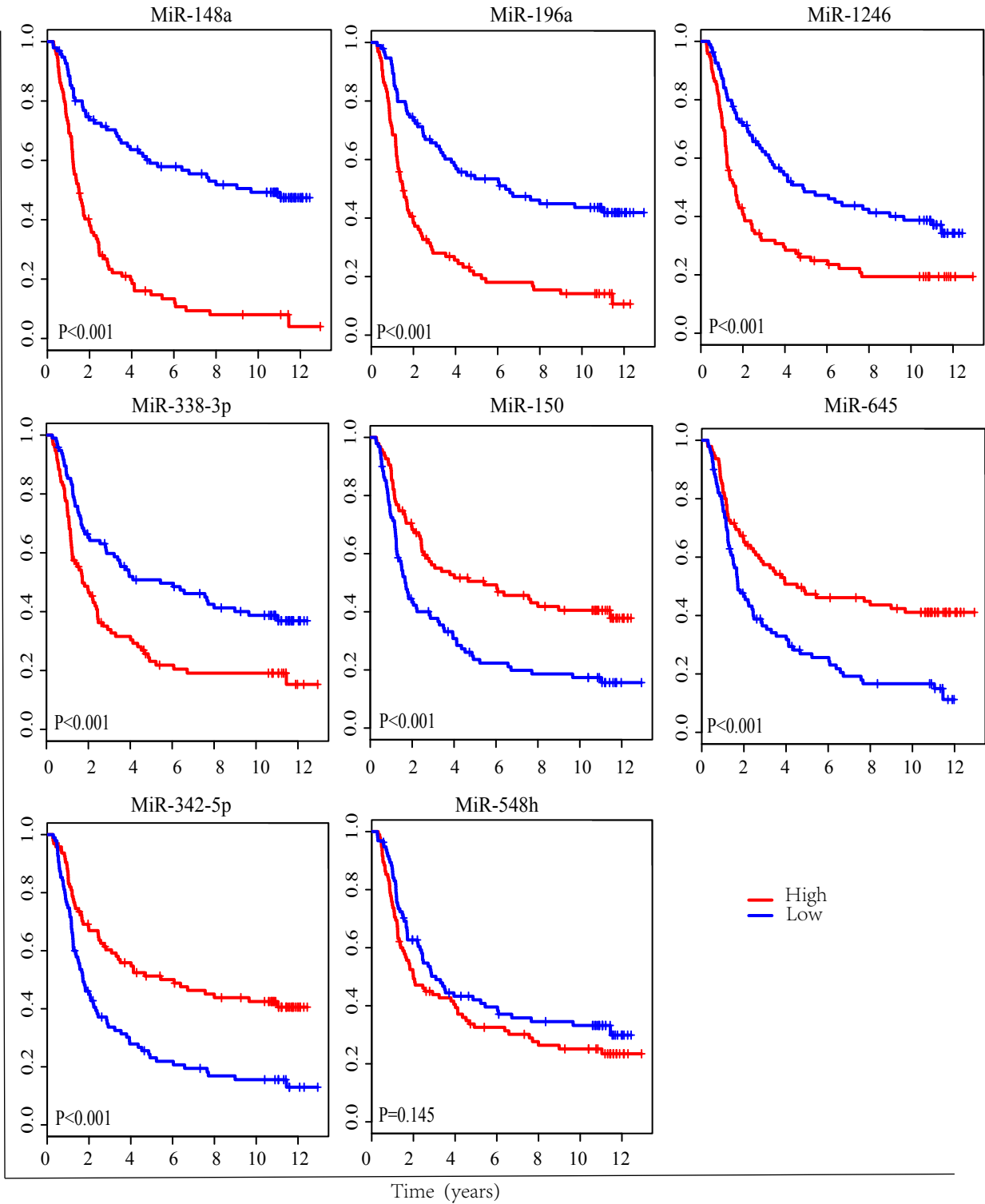

Supplement: Supplemental Information 6 [file peerj-08-9943-s006.pdf]

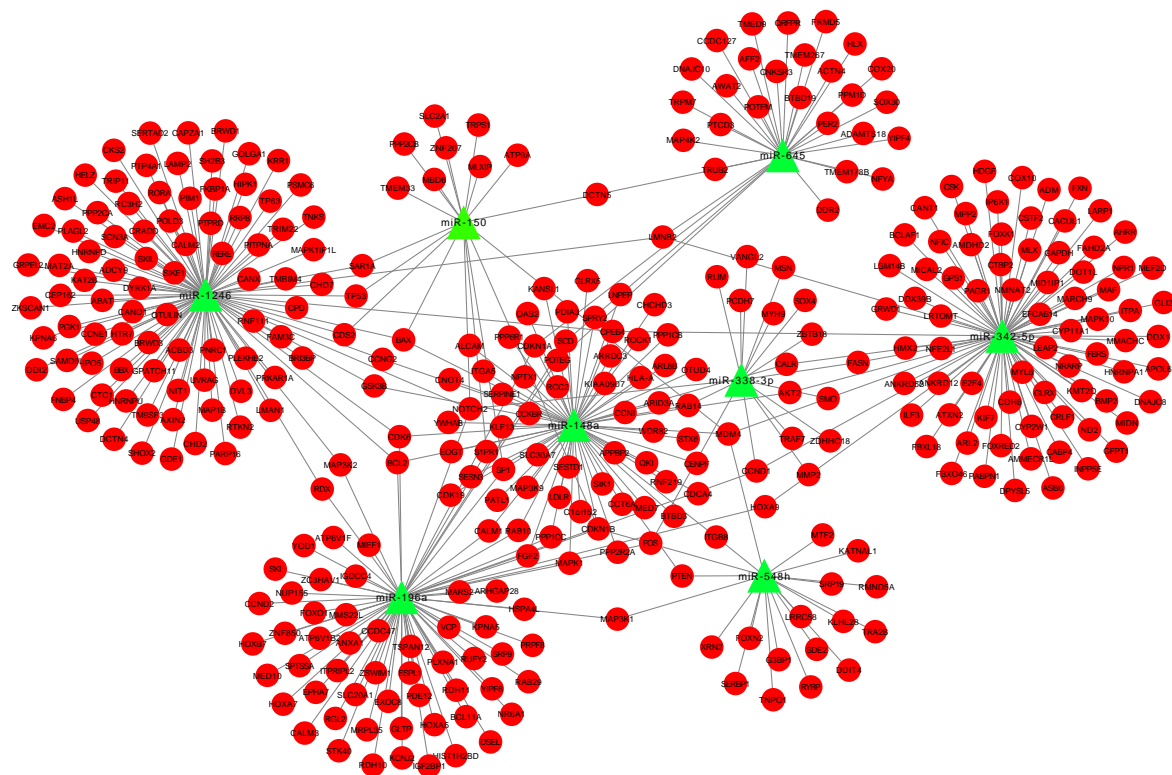

Supplement: Supplemental Information 7 — Green triangle and red node represent miRNA and target gene, respectively. [file peerj-08-9943-s007.pdf]
